# Lymphoid Organ-Resident Dendritic Cells Exhibit Unique Transcriptional Fingerprints Based on Subset and Site

**DOI:** 10.1371/journal.pone.0023921

**Published:** 2011-08-19

**Authors:** Kutlu G. Elpek, Angelique Bellemare-Pelletier, Deepali Malhotra, Erika D. Reynoso, Veronika Lukacs-Kornek, Rosemarie H. DeKruyff, Shannon J. Turley

**Affiliations:** 1 Department of Cancer Immunology and AIDS, Dana Farber Cancer Institute, Boston, Massachusetts, United States of America; 2 Division of Medical Sciences, Harvard Medical School, Boston, Massachusetts, United States of America; 3 Division of Immunology, Children's Hospital Boston, Harvard Medical School, Boston, Massachusetts, United States of America; 4 Department of Microbiology and Immunobiology, Harvard Medical School, Boston, Massachusetts, United States of America; Institut Pasteur, France

## Abstract

Lymphoid organ-resident DC subsets are thought to play unique roles in determining the fate of T cell responses. Recent studies focusing on a single lymphoid organ identified molecular pathways that are differentially operative in each DC subset and led to the assumption that a given DC subset would more or less exhibit the same genomic and functional profiles throughout the body. Whether the local milieu in different anatomical sites can also influence the transcriptome of DC subsets has remained largely unexplored. Here, we interrogated the transcriptional relationships between lymphoid organ-resident DC subsets from spleen, gut- and skin-draining lymph nodes, and thymus of C57BL/6 mice. For this purpose, major resident DC subsets including CD4 and CD8 DCs were sorted at high purity and gene expression profiles were compared using microarray analysis. This investigation revealed that lymphoid organ-resident DC subsets exhibit divergent genomic programs across lymphoid organs. Interestingly, we also found that transcriptional and biochemical properties of a given DC subset can differ between lymphoid organs for lymphoid organ-resident DC subsets, but not plasmacytoid DCs, suggesting that determinants of the tissue milieu program resident DCs for essential site-specific functions.

## Introduction

Dendritic cells (DCs) are present throughout the body and function as immune sentinels by capturing antigens and detecting danger signals from their surroundings. This information is then integrated to either promote T cell immunity or tolerance [1], [2], [3]. DCs are broadly classified as lymphoid organ-resident or migratory [2], [3]. The major subsets of secondary lymphoid organ-resident DC in mice include CD8 DCs (CD11c^high^CD11b^−^CD8^+^CD4^−^) and CD8^-^ DCs that can be further divided into CD4 DCs (CD11c^high^CD11b^+^CD8^−^CD4^+^) and double/triple negative DCs (CD11c^high^CD11b^+/−^CD8^−^CD4^−^) [2], [3]. While CD8, CD4 and CD4^−^CD8^−^ DCs are resident in all secondary lymphoid organs, only CD8 DCs are resident in the thymus.

The development of different DC subsets is controlled by specific transcription factors. For example, CD8 DCs are absent or reduced in mice lacking IRF8, Id2 and Batf3 [4], [5], [6] whereas CD8^−^ DCs are absent or reduced in mice deficient for IRF2 and IRF4 [7], [8]. Some of these transcription factors control development of additional DC subsets, as IRF8-deficient mice exhibit a marked reduction in plasmacytoid DCs (pDCs) [6], [9], and Batf3-deficient mice lack the migratory CD103^+^ DCs in skin, intestine, and lung [10]. Beyond developmental differences, important functional differences have been observed among DC subsets. For example, even though all DCs efficiently process and present antigens to T cells, CD8 DCs are specialized for cross-presenting exogenous antigens via MHC class I to CD8 T cells [11], [12], [13], whereas CD8^−^ DCs are superior in antigen presentation to CD4 T cells [11]. Furthermore, recent studies using expression profiling and proteomics demonstrated that a number of gene products related to antigen presentation are differentially expressed between CD8 and CD8^−^ DCs in the spleen [11], [14], [15]. The dichotomy between DC subsets has become widely accepted as a paradigm for all lymphoid organ-resident DCs. However direct experimental evidence to support this model and mechanistic data to explain their functional proclivities are lacking.

Although transcriptional and functional relationships between various secondary lymphoid organ-resident DC subsets at steady-state have been described, the relevant studies focused on subsets isolated from a single lymphoid organ, spleen [11], [14], [15], [16], [17]. Phenotypically, spleen and lymph nodes contain the same resident DC subsets [2], [3]. However, each site is physiologically different, and thus, the individual microenvironment of each site may influence their development and function. For example, splenic DCs are exposed to blood-borne molecules, while DCs in mesenteric lymph nodes are constantly exposed to intestine-derived antigens and signals. Thus, the genomic association among DC subsets from different lymphoid organs and possible differences due to microenvironment-derived factors has remained enigmatic.

To address this issue, we performed genome-wide expression analysis, creating a unique transcriptional fingerprint for each resident DC subset in spleen, skin-draining lymph nodes, mesenteric lymph nodes, and thymus of C57BL/6 mice. We successfully identified and characterized a signature gene expression profile relevant to major lymphoid organ-resident DC subsets, regardless of location. This allowed us to create a broadly applicable, subset-specific schema for division of labor among these subsets in any lymphoid organ. Strikingly, our analysis also revealed that each lymphoid tissue may separately imprint their resident DCs with a characteristic gene expression program, thereby influencing DC function. This held true even for such similar tissues as skin-draining and mesenteric lymph nodes, which each imposed distinct transcriptional profiles on resident DCs. In addition, thymic CD8 DCs exhibited high variation compared to CD8 DCs from secondary lymphoid organs, whereas, pDCs exhibited only minor differences across secondary lymphoid organs. By comparing and contrasting resident DCs according to surface phenotype as well as location, these data represent the largest comparative transcriptional study of lymphoid organ-resident DCs. Our results reveal a previously unappreciated level of site-specific specialization among DCs, while extending current theory regarding lineage relationships between CD8, CD4 and other DC subsets.

## Results

### Genomic divergence among CD8 DCs, CD4 DCs and pDCs spans primary and secondary lymphoid organs

Previous studies, focusing only on spleen, suggested a division of labor between murine CD8 and CD4 DCs [11], [14], [15], [16], [17]. However, it had not been previously determined whether and to what extent this functional dichotomy existed across multiple lymphoid organs. To this end, the genome-wide transcriptional relationships between resident DC subsets from multiple lymphoid organs were evaluated by sorting CD8 DCs from four sites: spleen, skin-draining lymph nodes (SLN), gut-draining mesenteric lymph nodes (MLN) (**Figure S1A**) and thymus (**Figure S1B**), and CD4 DCs from three sites: spleen, MLN, and SLN (**Figure S1A**). While secondary lymphoid organ-resident DCs represent cells that complete their differentiation and life history within the microenvironment of a single lymphoid organ, pDCs represent cells that travel from one lymphoid organ to another after their development in bone marrow [18]. Therefore, as a comparison, pDCs were also sorted from spleen, SLN and MLN (**Figure S1C**). The gene expression profiles of all DC subsets were analyzed using Affymetrix mouse Gene ST 1.0 chips.

**Figure 1:**
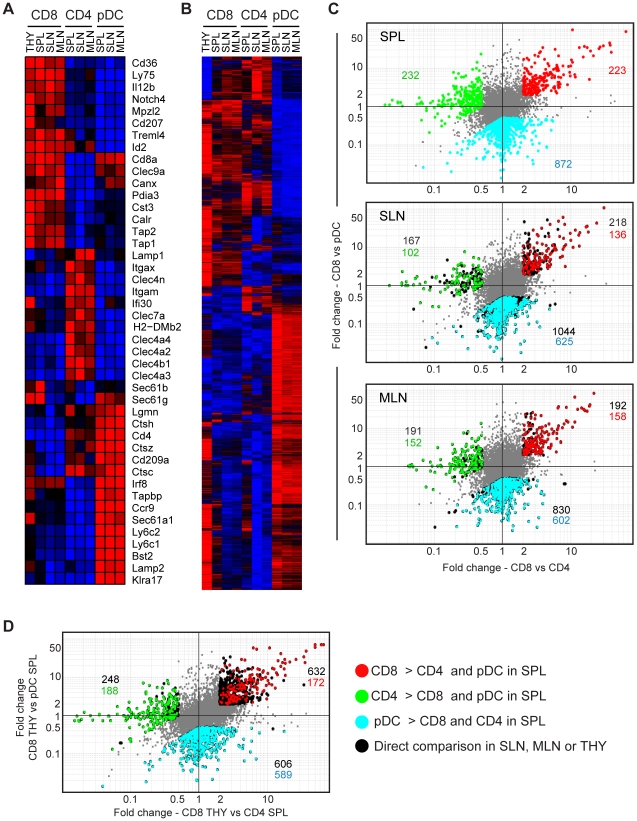
Genomic divergence between CD8 DCs, CD4 DCs and pDCs across lymphoid organs. (A) Signature gene expression profiles of CD8 DCs, CD4 DCs and pDCs in spleen (SPL), SLN and MLN, and CD8 DCs in thymus (THY) were analyzed by hierarchical clustering and visualized as a heatmap. Expression level: red>black>blue. (B) Hierarchical clustering of CD8 DCs, CD4 DCs and pDCs after selection of genes by the following criteria: >2-fold difference in any combination of populations, coefficient of variation (CV)<0.5 for each population, and mean expression >120 for at least one population. Expression level: red>black>blue. (C) Comparison of gene expression profiles of DC subsets in fold change (x-axis, CD8 vs CD4) vs fold change (y-axis, CD8 vs pDC) plots. Probes with at least 2-fold difference in expression for one subset compared to the other two in spleen (top), SLN (middle) and MLN (bottom) are identified (CV<0.5 for each CD8 DC, CD4 DC and pDC population; mean expression >120 in at least one population). In the spleen, probes that are >2-fold differentially expressed are highlighted in red (CD8 DCs), green (CD4 DCs) and cyan (pDCs). In SLN and MLN, probes with fold changes >2 are highlighted in black, and probes from the splenic analysis that are also at least 2-fold differentially expressed in SLN and MLN are highlighted in matching colors. Numbers of probes are indicated on the plots in matching colors. (D) Probes above the 2-fold cutoff associated with thymic CD8 DCs, splenic CD4 DCs and pDCs are shown in black on a fold change (x-axis, CD8 THY vs CD4 SPL) vs fold change (y-axis, CD8 THY vs pDC SPL) plot. Probes associated with splenic CD8, CD4 DCs and pDCs identified in Figure 1C are highlighted in matching colors. Numbers of probes are indicated on the plot with matching colors.

First, the transcriptional divergence between CD8 DCs, CD4 DCs and pDCs was evaluated using a list of 43 genes representing subset-associated ‘signature genes’ based on previous reports [11], [14], [16], [19], [20], [21]. In spleen, the DC subsets exhibited the expected gene expression profiles (**Figure 1A**) as levels of *Cd36* and *Ly75* (DEC205) were high in CD8 DCs compared to both CD4 DCs and pDCs, whereas those of *Clec4a4* (DCIR2) and *Clec4a2* (DCIR1) were higher in the CD4 subset. As reported previously, both CD8 DCs and pDCs expressed high levels of *Clec9a* (DNGR1) compared to CD4 DCs whereas *Bst2* (PDCA-1), *Ccr9* and *Klra17* (Ly-49Q) were expressed at high levels exclusively by pDCs [19], [20], [21]. Notably, the pattern of subset-associated canonical gene expression observed in splenic DCs was similar in the equivalent DCs from MLN and SLN (**Figure 1A**, and **Table S1**).

Next, a much broader analysis of gene expression by CD8 DCs, CD4 DCs and pDCs from different lymphoid organs was carried out. For this analysis, all 25108 probes (21968 genes) on the chip were analyzed and those with a fold-change cutoff of >2 in at least one population compared to any other (coefficient of variance (CV)<0.5 for each, mean expression >120 in at least one population) were considered to be differentially expressed and selected for further evaluation. Using these criteria, 6383 probes (5646 genes) were identified and analyzed by hierarchical clustering (**Figure 1B**). Heatmap visualization revealed clusters of genes enriched in each DC subset across multiple lymphoid organs. As shown in a fold change plot for spleen, 223 probes (208 genes) were found to be more highly expressed in CD8 DCs compared to CD4 DCs and pDCs, 232 probes (215 genes) were more highly expressed in CD4 DCs compared to CD8 DCs and pDCs, and 872 probes (810 genes) were more highly expressed in pDCs compared to CD8 DCs and CD4 DCs (**Figure 1C**, top). Performing the same analysis for DC subsets from SLN and MLN indicated that a similar degree of inter-population divergence existed in those sites (**Figure 1C**, middle and bottom, black).

To ascertain whether the specific inter-population differences were shared across secondary lymphoid organs, the differentially expressed genes identified among splenic DC subsets were interrogated in the corresponding subsets from SLN and MLN (**Figure 1C** **,** middle and bottom). Of the splenic CD8 DC-associated probes, ∼60–70% were also >2-fold more highly expressed by CD8 DCs in lymph nodes compared with CD4 DC and pDC (**Figure 1C**, red). For CD4 DCs, 45–65% of the splenic probes were also more highly expressed in the lymph node CD4 DC cohort compared with CD8 DCs or pDCs (**Figure 1C**, green). Finally, ∼70% of the probes more highly expressed by splenic pDCs compared with CD8 and CD4 DCs were also differentially expressed by pDCs from lymph nodes (**Figure 1C**, cyan).

**Figure 2:**
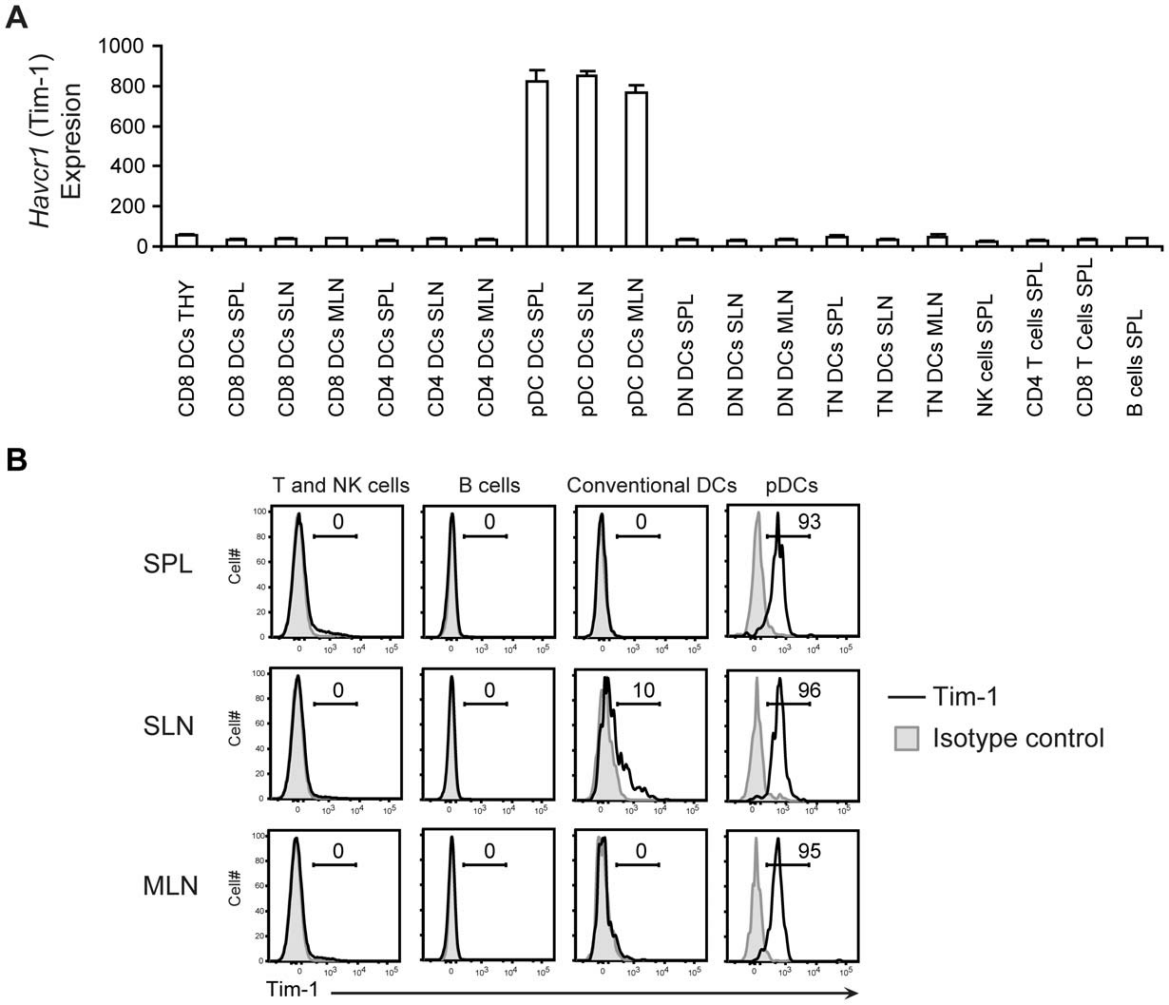
Expression of TIM-1 by pDCs from secondary lymphoid organs. (A) Bar graph showing the expression value of *Havcr1* gene for CD8 DCs, CD4 DCs, DN DCs, TN DCs and pDCs from different lymphoid organs and naïve NK cells, CD4 T cells, CD8 T cells and B cells from spleen (data obtained from ImmGen). (B) Various immune cell populations from spleen, SLN and MLN were analyzed for surface Tim-1 expression using flow cytometry: T and NK cells (gated on mixed CD19^−^CD3ε^+^ cells and CD19^−^NK1.1^+^ cells), B cells (gated on CD3ε^−^NK1.1^−^CD19^+^B220^+^ cells), conventional DCs (gated on CD3ε^−^CD19^−^NK1.1^−^B220^−^CD11c^high^ cells), and pDCs (gated on CD3ε^−^CD19^−^NK1.1^−^B220^+^PDCA-1^+^CD11c^low^ cells). Gray filled, isotype control; black line, anti-Tim-1. Representative histograms from at least 3 different experiments are shown.

As shown in previous studies, genes such as *Ly75*, *Cd86*, *Tlr3*, and *Tlr11* were associated with CD8 DCs, *Clec4a4* was associated with CD4 DCs, *Ifnar1/2*, *Tlr7* and *Tlr9* were associated with pDCs in all secondary lymphoid organs (**Table S2**). Importantly, a large number of genes that are new to the field and potentially important for DC function or development were identified. As examples, expression levels of the transcription factor *Met* (HGFR) and the phosphatase *Ppap2a* (phosphatidic acid phosphatase type 2A) were higher in CD8 DCs across secondary lymphoid organs whereas immunoregulatory *S100A4* (S100 calcium binding protein A4), and *Nod1* were more highly expressed in CD4 DCs. Genes such as *Duxbl* (double homeobox B-like), *Havcr1* (Tim-1), immunoregulatory *Sema4b* (Semaphorin 4B) and *Slamf9* (SLAM family member 9) were more highly expressed in pDCs across secondary lymphoid organs. Of note, several of these newly identified subset-associated genes, such as *Met*, *Duxbl*, *Ppap2a*, *Dscam* and *Slamf9*, were found to be strikingly DC- or myeloid-specific when compared to key leukocyte populations such as B cells, T cells and NK cells (**Figure S2**). Interestingly, flow cytometry analysis indicated that Tim-1, which is inducible on T helper 2 cells [22], is expressed exclusively by pDCs in secondary lymphoid organs at steady-state (**Figure 2**).

Having identified subset-associated genomic signatures among secondary lymphoid organ-resident DCs, we next sought to depict the relationships between DCs that reside in primary and secondary lymphoid organs. The thymus contains a sizeable population of CD8 DCs compared with secondary lymphoid organs (Ref. [23] and **Figure S1B**) however their precise function remains to be elucidated. Therefore, comparing the transcriptome of thymic CD8 DCs with DC subsets in other lymphoid organs may shed new light on their long sought after function. Because thymus is largely devoid of CD4 DCs, thymic CD8 DCs were compared to CD4 DCs and pDCs from spleen. Thymic CD8 DCs expressed the CD8 DC-associated signature genes in a similar manner as their counterparts in secondary lymphoid organs (**Figure 1A**). Based on this observation, the probes identified as splenic CD8 DC-associated in Figure 1C were then used to compare thymic CD8 DCs with CD4 DCs and pDCs from secondary lymphoid organs. Consistent with splenic CD8 DCs, thymic CD8 DCs exhibited a large number of transcriptomic differences with CD4 DCs and pDCs from all secondary lymphoid organs (**Figure 1D**).

Overall, these data indicate that transcriptional divergence between CD8 DCs, CD4 DCs and pDCs is largely conserved across primary and secondary lymphoid organs and illuminate new DC subset-associated genes for future research. Embedded within these data are the conserved genomic roadmaps that endow CD8 DCs, CD4 DCs and pDCs with attributes essential to their core functions across secondary lymphoid organs.

### Site-specific transcriptional fingerprints among lymphoid organ-resident DCs

With the genomic and proteomic profiles of CD8 DCs and CD4 DCs having only been established for spleen [11], [14], [15], [16], [17], it has remained unclear to what extent a given DC subset from one lymphoid organ relates to the same subset residing in another lymphoid organ. Therefore, we sought to determine whether the transcriptomic signatures of each resident DC subset were conserved across lymphoid organs from distant anatomic locales.

**Figure 3:**
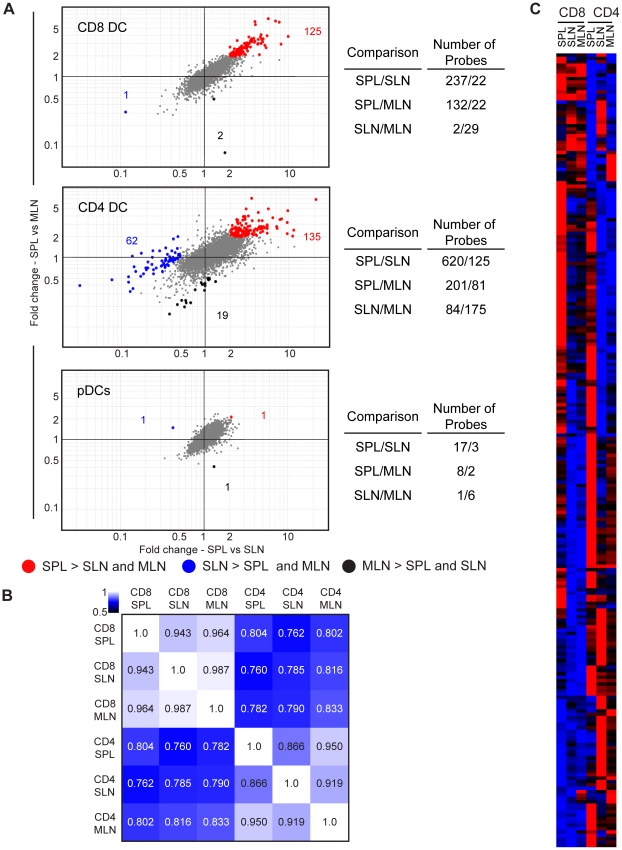
Identification of secondary lymphoid organ-specific differences among DCs subsets. (A) Left, Gene expression profiles of CD8 DCs (top), CD4 DCs (middle) and pDCs (bottom) in the spleen, SLN and MLN were compared and probes above the 2-fold cutoff in one organ compared to the other two are highlighted on fold change (x-axis, SPL vs SLN) vs fold change (y-axis, SPL vs MLN) plots. Numbers of probes are indicated on the plots. Right, numbers of probes above 2-fold difference in expression for organ pairs are summarized in tables for CD8 DCs (top), CD4 DCs (middle) and pDCs (bottom). Probe selection criteria: CV<0.5 in each population of a given subset, mean expression >120 for at least one population of a given subset. Red, SPL >SLN and MLN; blue, SLN>SPL and MLN; black, MLN>SPL and SLN. (B) Correlation matrix comparison of CD8 DCs and CD4 DCs across the spleen, SLN and MLN. Numbers indicate correlation coefficient. (C) A dataset including all the genes identified in Figure 3A for CD8 and CD4 DCs are analyzed by hierarchical clustering and illustrated using a heatmap. Expression level: red>black>blue.

The gene expression profiles of CD8 DCs, CD4 DCs or pDCs were compared in a pair-wise fashion: Spleen vs SLN and MLN, SLN vs spleen and MLN, and MLN vs spleen and SLN using an fold-change cutoff value of 2 (CV<0.5 for each, mean expression >120 in at least one population) (**Figure 3A**, left). This analysis pointed to several major outcomes. First, both CD4 and CD8 DCs exhibited transcriptomic differences specific to location. For example, 125 probes (89 genes) were more highly expressed by splenic CD8 DCs compared to their lymph node counterparts, and 135 probes (113 genes) were more highly expressed by splenic CD4 DCs compared to the lymph node equivalents (**Table S3**). Second, DCs that were resident in SLN and MLN were more similar to each other than to spleen-resident DCs. For CD4 DCs, 62 probes (60 genes) and 19 probes (19 genes) were associated with SLN or MLN, respectively, whereas CD8 DCs exhibited few differences between MLN and SLN (**Table S3**). Indeed, the differences between CD4 DCs across secondary lymphoid organs were greater than the differences between CD8 DCs when probe numbers were identified by single organ-to-organ comparisons as tabulated in **Figure 3A** (right). For example, 2-3x more probes differed between splenic and SLN CD4 DCs compared to CD8 DCs from spleen and SLN (620 vs 237). Interestingly, and in contrast to the lymphoid-organ resident DCs, pDC expression profiles were almost identical across secondary lymphoid organs (**Figure 3A**, bottom).

Next we evaluated the degree of similarity among all CD4 and CD8 DC populations interrogated in this study. As shown in the correlation matrix, each of the CD4 DC populations were clearly more similar to one another than to the CD8 DC populations, however, site-specific variation among each set of populations was also apparent (**Figure 3B** **).** Hierarchical clustering of the differentially expressed genes shown in Figure 3A revealed clusters of transcripts that tracked with a single lymphoid organ for CD4 and CD8 DCs (**Figure 3C**). These belonged to a wide range of functional groups including inhibitory/activating receptors and ligands, adhesion and signaling molecules, solute carriers, and ion transporters. For instance, *hmox1*, which encodes the immunomodulatory enzyme, heme oxygenase I [24], was enriched in splenic CD8 DCs with ∼4-fold higher expression compared to lymph node CD8 DCs and ∼3-fold higher compared to all CD4 DCs (**Table S4**).

**Figure 4:**
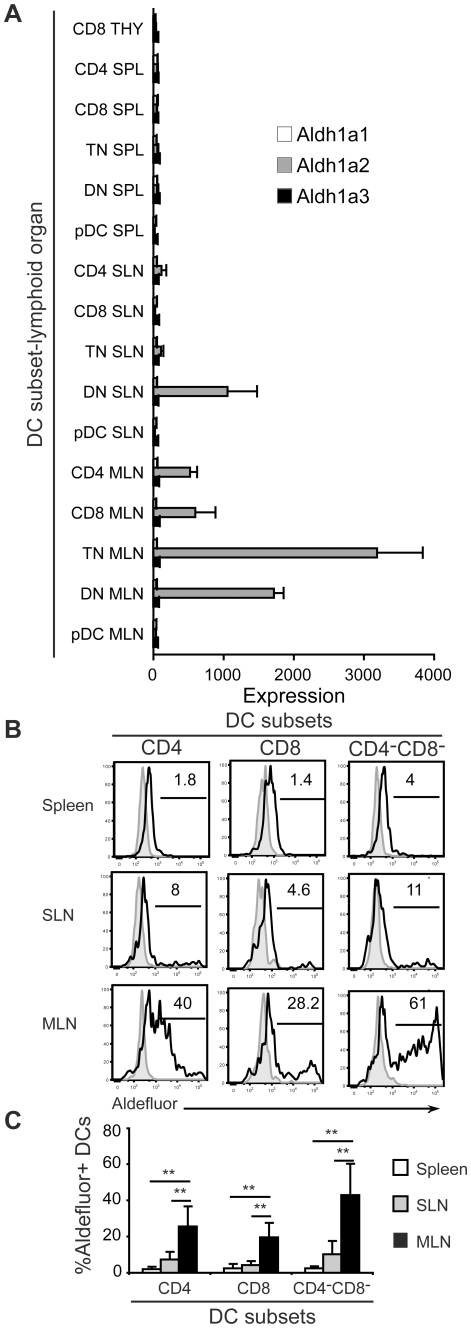
Aldh1a2 expression is a shared property among DC subsets in MLNs. (A) Mean expression values for *Aldh1a1* (white), *Aldh1a2* (gray) and *Aldh1a3* (black) genes by CD4, CD8, CD4^−^CD8^−^CD11b^+^ double negative (DN), CD4^−^CD8^−^CD11b^−^ triple negative (TN) DCs and pDCs in secondary lymphoid organs and CD8 DCs in the thymus. (B) Representative histograms showing aldehyde dehydrogenase activity based on Aldefluor staining in DC subsets. Numbers indicate the percentage of Aldefluor^+^ cells. Black line, Aldefluor staining; gray-filled, Aldefluor staining in the presence of the aldhehyde dehydrogenase inhibitor DEAB. (C) Bar graphs showing the percentage of Aldefluor^+^ DCs in secondary lymphoid organs. Data are representative of 3–4 independent experiments. ** indicates P<0.01. Data are represented as mean+SD.

Among the genes expressed by DCs in the MLN is *Aldh1a2* (Aldhehyde dehydrogenase 2), also known as retinal dehydrogenase 2 (RALDH2). Aldh1a2, with two other enzymes Aldh1a1 and Aldh1a3, function in retinol metabolism, converting retinal into retinoic acid (RA) [25]. RA has been implicated in immune pathways in the gut environment including the imprinting of T and B cells with intestinal tropism [26], [27] and the local generation of regulatory T cells [28]. Recent studies have suggested that the RALDHs involved in RA generation are selectively expressed by CD103^+^ intestinal DCs [29] and MLN-resident stromal cells [30]. However, little is known about the role of lymph node-resident DC subsets in RA generation. The expression levels of *Aldh1a1*, *Aldh1a2* and *Aldh1a3* were compared among CD4, CD8, CD4^−^CD8^−^CD11b^+^ double negative (DN) and CD4^−^CD8^−^CD11b^−^ triple negative (TN) DCs (sorting strategy in **Figure S1A**) as well as CD8 DCs from thymus, and pDCs from spleen, SLN and MLN. The signal for *Aldh1a2* was detected in all DC populations from MLN except pDCs, and expressed in DN DCs from SLN (**Figure 4A**, gray bars). However, *Aldh1a1* and *Aldh1a3* were not expressed by any DCs subset studied here (**Figure 4A**, white and black bars). Next, the flow cytometry-based Aldefluor assay was used to determine whether the Aldh1a2 enzyme was active in these DC subsets. Given that *Aldh1a1* and *Aldh1a3* are not expressed by DCs, any enzyme activity detected in this assay would be attributable to Aldh1a2. Little or no enzyme activity was detected in splenic and SLN-resident DC subsets whereas CD4, CD8 and CD4^−^CD8^−^ DCs from MLN were Aldefluor^+^ (**Figure 4B and C**). There was a tight correlation between *Aldh1a2* gene expression levels (**Figure 4A**) and the proportion of Aldefluor^+^ DCs (**Figure 4C**). Taken together, our results demonstrate that all subsets of conventional DCs in MLNs but not pDCs express *Aldh1a2* and functional Aldh1a2 enzyme. That multiple conventional DC subsets in a given secondary lymphoid organ can display identical site-specific functions at both the transcriptional and protein levels whereas pDCs exhibit relatively few site-specific differences suggests that conventional DCs are more prone to programming by their immediate surroundings, at least under steady state conditions. Programming of DC function by microenvironmental cues might ensure tasks critical for the particular anatomical site in which they reside.

### Significant divergence between thymic and secondary lymphoid organ-resident CD8 DCs

**Figure 5:**
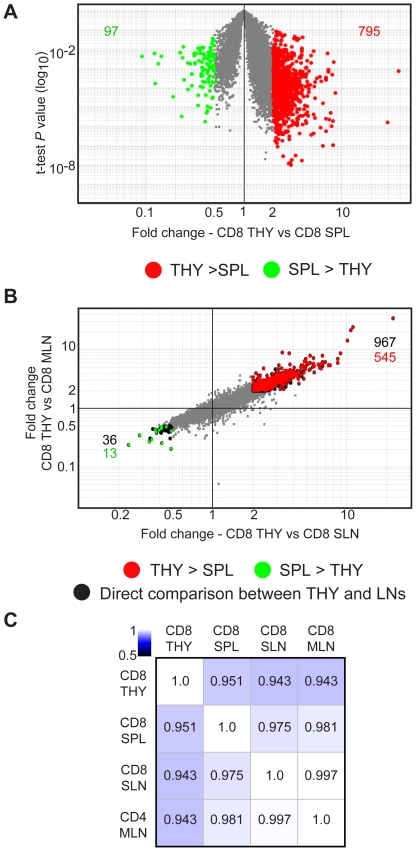
Comparison of gene expression profiles of CD8 DCs in the thymus and secondary lymphoid organs. (A) Fold change (x-axis, CD8 THY vs SPL) vs T-test P value (log10, y-axis, CD8 THY vs SPL) plot showing the number of probes with a difference in expression of at least 2-fold (CV<0.5 for each CD8 population; expression >120 for at least one population). Red, CD8 THY>SPL; green, CD8 SPL>THY. (B) Gene expression profile of CD8 DCs in the thymus compared with SLN and MLN. >2-fold differentially expressed probes in the thymus relative to both SLN and MLN are highlighted on a fold change (x-axis, CD8 THY vs CD8 SLN) vs fold change (y-axis, CD8 THY vs CD8 MLN) plot. Probes identified in Figure 5A that remain above the 2-fold cutoff in these other sites are highlighted in matching colors. Numbers of probes are indicated on the plot in matching colors. (C) Correlation matrix for CD8 DCs across the thymus, spleen, SLN and MLN. Numbers indicate correlation coefficient.

Given the variation observed in gene expression among spleen and lymph node-resident CD8 DCs, we next sought to determine whether a similar degree of divergence existed between CD8 DCs from primary and secondary lymphoid organs. From the heatmap in Figure 1B, it was evident that clusters of genes were enriched in thymic CD8 DCs compared with secondary lymphoid organ-resident CD8 DCs. To evaluate this further, a direct comparison of the genome-wide expression profiles of thymic CD8 DCs with secondary lymphoid organ-resident CD8 DCs was performed. The transcriptomic relationship between the thymic and splenic CD8 DCs was then examined by analyzing the distribution of differentially expressed genes on a fold change vs t-test P value plot. Using a 2-fold cutoff (CV<0.5 for each, mean expression >120 for at least one population), 795 probes (725 genes) were found to be more highly expressed by thymic CD8 DCs compared with splenic CD8 DCs (**Figure 5A** and **Table S5**). On the other hand, 97 probes (95 genes) were associated with splenic CD8 DCs compared with their thymic counterparts (**Figure 5A** and **Table S5**). When, thymic CD8 DCs were compared to lymph node CD8 DCs, we found that 967 probes (803 genes) were more highly expressed by thymic CD8 DCs, whereas 36 probes (34 genes) were associated with lymph node CD8 DCs compared to thymic CD8 DCs (**Figure 5B**, black). Next, the relative expression pattern of those genes differentially expressed among thymic and splenic CD8 DCs was evaluated in the corresponding subsets from SLN and MLN (**Figure 5B**) using a fold change (thymus vs SLN) vs fold change plot (thymus vs MLN). We found that 545 of the 795 probes (537 genes) were also more highly expressed by thymic CD8 DCs compared with CD8 DCs from lymph nodes (**Figure 5B**, red) whereas, 13 out of 97 probes (13 genes) were more highly expressed in CD8 DCs from both lymph nodes compared to thymic CD8 DCs (**Figure 5B**, green).

These results demonstrated that a large number of genes are more highly expressed by thymic CD8 DCs compared to their counterpart in secondary lymphoid organs. This is further supported by data shown in the correlation matrix, which indicates that CD8 DCs in secondary lymphoid organs are more similar to one another than to their thymic counterparts (**Figure 5C**). In sum, our analysis of thymus- and secondary lymphoid organ-resident DCs not only extends the breadth of the transcriptional signature for CD8 DCs but also illuminates hitherto unrecognized differences in gene expression between lymphoid microenvironments.

### Subset and location shape transcriptional fingerprints of lymphoid organ-resident DCs

**Figure 6:**
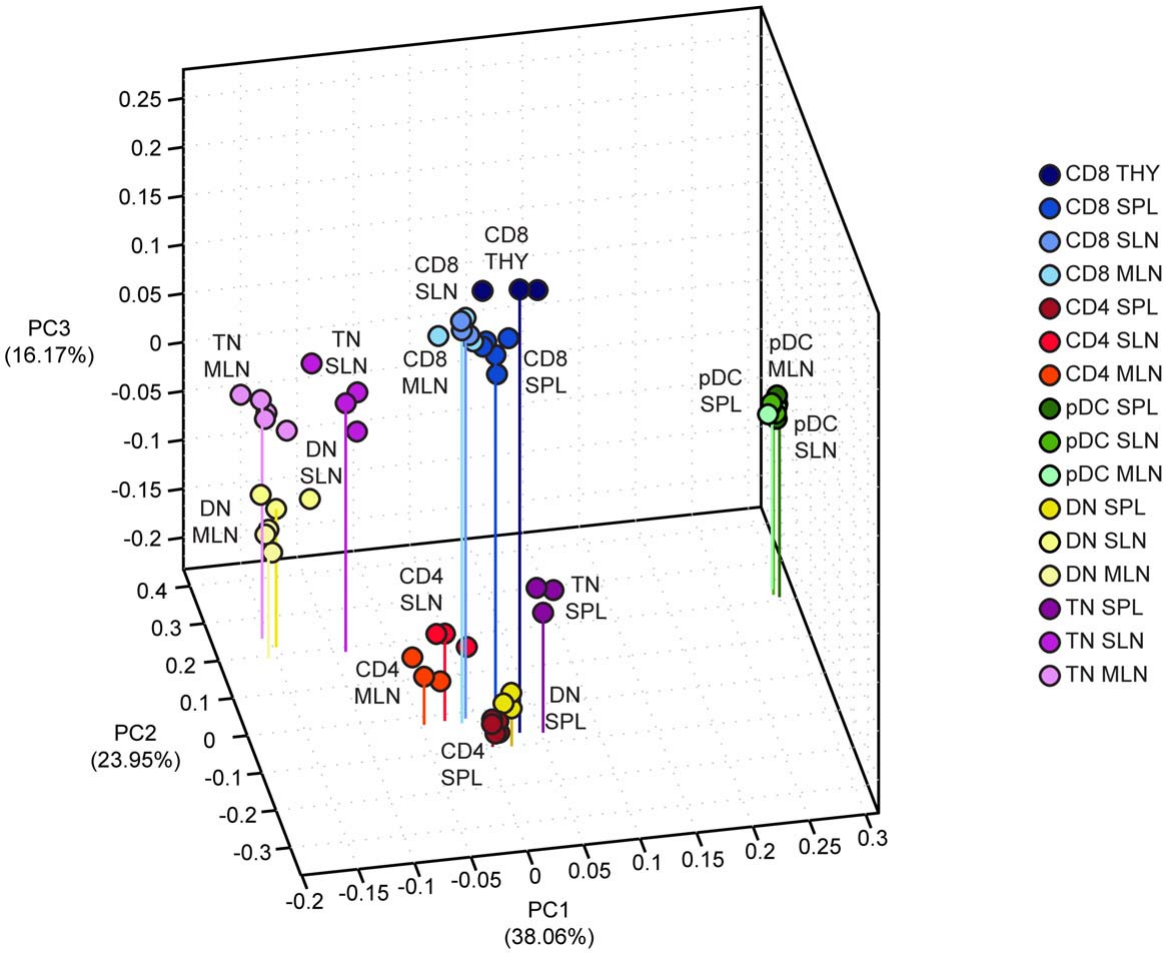
Global relationships among major subsets of lymphoid organ-resident DCs. Principal component analysis comparing DC subsets from different lymphoid organs. Percentages reflect the total variation among populations in each principal component (PC). Each circle in the 3D plot indicates a replicate for the 16 DC subsets. Lines indicate the coordinates of one representative replicate for each subset on PC1 and PC2.

Next, the relative transcriptomic relationships between CD8 DCs, CD4 DCs and pDCs with respect to other DCs subsets across primary and secondary lymphoid organs were evaluated. To this end, principal component analysis (PCA) was used to measure of population distance based on inter-subset variation (**Figure 6**). In this analysis, we included DN and TN DCs from spleen (resident DCs), SLN and MLN (mixture of resident and migratory DCs), CD4, CD8 and pDCs from secondary lymphoid organs, and CD8 DCs from thymus, and individual replicates for each population were plotted. The largest variation measured among the subsets existed between pDCs and conventional DCs. pDCs across secondary lymphoid organs clustered tightly, indicating high level of similarities in their transcriptional profile regardless of location. Conventional DCs were characterized by distinct clusters: CD8 DCs from secondary lymphoid organs and thymus, CD4 DCs from secondary lymphoid organs closely associated with DN/TN DCs from spleen, and finally, migratory DC-rich DN/TN DCs from lymph nodes. Consistent with our results, the PCA also measured significant intra-population differences for all subsets except pDCs, though these were smaller than the inter-population distances. Thus, the relative distance between DC subsets supports the notion that lymphoid organ-resident DCs and pDCs, as well as DN and TN DCs, differ rather distinctly from each other at the transcriptomic level.

## Discussion

Conventional DCs are dedicated antigen presenting cells that orchestrate T cell responses [2], [3]. Compared with other leukocytes, DCs are specialized for efficient antigen capture, processing and presentation. Likewise, they are armed with a variety of receptors that allows them to survey their microenvironment and integrate inflammatory cues including pathogen and damage associated signals [31]. Emerging studies suggest that DC subsets of distinct developmental origin cooperate with one another in a functional sense but carry out different aspects of adaptive immunity. Among lymphoid-organ DCs, division of labor has been analyzed most extensively, using transcriptional profiling and proteomic assays, in splenic CD8 and CD8^−^ DCs due to their abundance and accessibility [11], [14], [15], [16]. Other studies focused on the relative role of resident and migratory DC subsets in T cell responses using mainly functional assay [10], [32], [33], [34], [35], [36]. Observations from these studies raised the possibility that subset-specific molecular programs might account for their unique roles in adaptive immunity. However, definitive evidence to explain how individual DC subsets from different lymphoid organs function and contribute to host defense and tolerance is still lacking.

To date, it is assumed that the function of a given DC subset is dictated by developmental specifications and that DCs belonging to a given subset would more or less exhibit the same genomic and functional programs throughout the body. Here, we interrogated the transcriptional relationships between lymphoid organ-resident DC subsets, particularly CD8 and CD4 DCs, from multiple sites including primary and secondary lymphoid organs. This investigation allowed us to 1) define genomic programs that distinguish lymphoid organ-resident DC subsets from one another throughout the body and 2) identify transcriptional and functional properties of a given lymphoid organ-resident DC subset that vary between anatomical locations. We confirmed the inter- and intra-subset genomic relationships by hierarchical clustering as well as by detailed comparisons of genomic and functional characteristics of the relevant DCs.

The multiple lymphoid organ approach to compare gene expression profiles of CD8 and CD4 DC allowed us to identify subset-associated transcriptional and functional programs that support the notion of a division of labor. Previous gene expression studies had identified differences between CD8 and CD4 DCs primarily in the spleen [11], [14], [15], [16]. Differential expression of C-type lectins such as DEC205 and DCIR2 by CD8 and CD8^−^ DCs, respectively, are well-known examples [11]. Antigen delivery studies targeting these receptors suggest functional bias of CD8 DCs towards CD8 T cells and CD8^−^ DCs towards CD4 T cells. Furthermore, compared to CD4 DCs, CD8 DCs exhibit a transcriptional program that is characterized by genes biased towards CD8 T cell responses such as MHC class I-related molecules, IL-12, IL-15 and XCR1 [11], [37], [38], [39], [40]. Our study built an extensive expression profile for CD4 and CD8 DCs consisting of numerous genes that differ between the two subsets across different lymphoid organs. Importantly, we identified novel gene targets critical for various cellular and molecular processes of DCs that have not been previously studied including solute carriers, immunomodulatory and signaling molecules such as transcription factors, kinases, phosphatases.

When the gene expression profile of a given DC subset is compared across multiple lymphoid organs, marked site-associated differences are observed. It is unclear why such differences exist but several plausible explanations can be surmised. One possibility is that the genomic program of DCs is influenced by the organ in which it resides. In that sense, determinants of the local tissue milieu might significantly impact gene expression and downstream function of DCs. Programming of gene expression by the microenvironment could result in a tissue-associated signature reflected in all DCs living in the vicinity. The expression of aldehyde dehydrogenase 2 by resident and migratory DC subsets in MLNs is an example of such a tissue-associated signature. Previous studies showed that particularly migratory DCs in the gut use this enzyme for imprinting T cells [29]. Consistent with this, we observed the higher level of expression and enzyme activity in double/triple negative DCs that contain high proportion of migratory DCs. Interestingly, this tissue-associated expression pattern is also observed among non-hematopoietic stromal cells that reside in MLNs [30]. Currently it is unclear how MLN DCs are programmed to express this enzyme, although dietary agents including vitamin A have been implicated [41]. After their development from common progenitors in the bone marrow, DCs that travel to MLN or gut tissue may undergo further tissue-specific programming mediated by soluble factors derived from the intestine and/or lymphoid stroma. Whether microenvironment-specific programming of gene expression is evident in other immune cells remains to be determined.

Global analysis as well as subset-to-subset analysis indicated that DCs residing in lymph nodes are clustered together and relatively distant from their counterparts in spleen. This may be due to tissue-specific programming of splenic DC subsets by the metabolite-rich contents of the blood. Interestingly, we found larger differences among CD4 DCs in different secondary lymphoid organs compared to the differences in CD8 DCs. One explanation may be that CD4 DCs are more responsive to microenvironmental cues due to their biochemical makeup or their proximity to such signals. Another explanation may be that splenic CD4 DCs are more heterogenous compared to their counterparts in lymph nodes. We also compared CD4^−^CD8^−^ DCs to other DC lymphoid organ resident subsets. We found that splenic CD4 and CD4^−^CD8^−^ DCs are very similar to one another in terms of gene expression profiles. This close relationship was also suggested by previous reports [14], [15] and here we confirmed this via global analysis of their transcriptomes. On the other hand, CD4^−^CD8^−^ DCs from the lymph nodes clustered separately compared to all resident DC subsets. This may be associated with the heterogeneity of CD4^−^CD8^−^ populations in lymph nodes due to the presence of various migratory DC subsets [2], [3]. The developmental and functional differences of pDCs compared to conventional DCs is also reflected in the global analysis in which pDCs were the most distant to any other DC subset studied here. In addition, the differences in gene expression profiles among pDCs from different secondary lymphoid organs were minimal compared to conventional DCs. Unlike conventional DCs that reside in lymphoid or non-lymphoid organs, pDCs develop in bone marrow and circulate throughout lymphoid organs via blood [18]. The developmental stage when they are exposed to different lymphoid organs and the transit time through each lymphoid organ may not be suitable to influence their transcriptome.

While the importance of CD8 DCs in cross-presentation of antigens to CD8 T cells in secondary lymphoid organs is well studied and appreciated, their role in the thymus remains unclear. The composition of thymic DCs is markedly different from DCs in secondary lymphoid organs. The proportion of CD8 DCs is relatively low in secondary lymphoid organs, representing 10–25% of all DCs (Ref. [2], [3] and this study). In the thymus, however, three DC populations, resident CD8 DCs, migratory CD8^−^Sirpα^+^ DCs and pDCs, have been identified and a large portion of these cells is comprised of CD8 DCs (∼50–90%) (Ref. [23] and this study). The large contingent of CD8 DCs in this organ suggests that they play an important role in thymic processes. CD8^−^Sirpα^+^ DCs have recently been implicated in thymic selection since these migratory cells can capture and carry peripheral tissue antigens to thymus [35]. However it remains to be determined whether resident CD8 DCs have such roles in the T cell selection process. Several studies suggest that CD8 DCs in SLN can acquire viral antigens carried by migratory DCs and present these antigens to CD8 T cells [34], [42]. Therefore, there may be a similar interaction between thymic CD8 DCs and migratory Sirpα^+^ DCs in terms of antigen presentation.

Although all CD8 DCs have a common transcriptional program reflecting their function, both global and organ-to-organ analyses indicated that thymic CD8 DCs are distant from their counterparts in secondary lymphoid organs. Among these differences, expression of a variety of endocytic receptors including at higher levels was observed. CD8 DCs are highly efficient at capturing apoptotic cells and cross-presenting antigens associated with these cells [43], [44]. Although the receptors involved in apoptotic cell capture by CD8 DCs are yet to be identified, receptors such as DEC205, Clec9A, CD36 and Treml4 [16], [20], [45], [46], [47], [48] may be involved. Overall, CD8 DCs in the thymus are equipped with a variety of endocytic receptor compared to both CD4 and CD8 DCs in secondary lymphoid organs suggesting that these cells may be heavily involved in clearance of apoptotic cells, particularly T cells that failed selection. The divergence in transcriptional profiles between CD8 DCs in thymus and secondary lymphoid organs may be explained by the differences in their developmental origin. Thymic CD8 DCs reside in a structurally different organ that is not known to have a peripheral sentinel function as lymph nodes or spleen. Importantly, while the majority of DCs arise from a myeloid progenitor [18], thymic CD8 DCs as well as pDCs are thought to be derived from lymphoid lineages and may carry traces of pre-T cell genes [49].

A unique aspect of our study was the use of a strict experimental procedure implemented by the multi-institute collaboration known as the Immunological Genome Project (ImmGen). We utilized the highly standardized protocol established by ImmGen which strives to minimize sample variation between participating laboratories by employing common reagents, limited processing times, centralized RNA handling, and stringent normalization and quality control measures (Ref. [50] and www.immgen.org). This has proven to be a powerful and precise approach for examining transcriptional relationships among more than 200 murine leukocyte subsets and hematopoietic progenitors. Furthermore, similar approaches are currently being taken to systematically evaluate the relationships between mouse and human leukocytes.

In conclusion, this study has illuminated novel transcriptional relationships among major lymphoid organ-resident DC subsets. DCs function as decision makers for T cell responses. DC subsets exhibit differences in the ways they detect signals from their surroundings and interact with different T cell subsets. Therefore, elucidating how each DC subset functions is essential to understand how they promote T cell tolerance and immunity. This is the first study to demonstrate site- and subset-associated relationships between lymphoid-organ resident DCs. We showed that while DCs exhibit transcriptional programs that correlate with their developmental origin and phenotypic subset, anatomical location promotes divergence from this program to adjust for functions associated with the microenvironment. Global analysis of these cells relative to multiple immune cell subsets in the ImmGen Project will further provide new dimensions to our understanding of the immune system. This knowledge will be invaluable for identifying novel targets and developing efficacious immunotherapeutics.

## Materials and Methods

### Ethics statement

All animal work has been carried out in accordance with US National Institutes of Health guidelines. This study is reviewed and approved by the Dana Farber Cancer Institute, Animal Care and Use Committee (ACUC) (protocol IDs: 04-025 and 07–038).

### Mice

Male C57BL/6 mice of 6 weeks of age were purchased from The Jackson Laboratories. Animals were maintained under pathogen free condition in the Animal Facility of the Dana-Farber Cancer Institute.

### Cell preparation, sorting and flow cytometry

For each DC sorting, tissues from at least 3 mice were pooled. Spleen, SLN, MLN or thymus were gently disrupted by forceps and incubated for 15 min at 37°C in phenol-red free DMEM (Mediatech) containing 10 mM HEPES, 0.1 mg/ml DNase I (Invitrogen) and 0.28 U/ml Liberase Blendzyme III/DL (collagenase/neutral protease with very low to negligible levels of endotoxin, Roche). Single cell suspensions were subjected to RBC-lysis using ACK buffer (Lonza Biowhittaker) and transferred into depletion buffer (PBS, 2 mM EDTA and 2% FBS) containing FcR-blocking antibody (2.4G2, produced in house). CD19, CD3ε, Gr1, NK1.1 and TER-119 positive cells were depleted using anti-biotin MACS beads (Miltenyi). Cells were stained in FACS buffer with combinations of fluorochrome-conjugated antibodies against CD11b (M1/70), CD4 (L3T4), CD11c (N418), CD8 (53–6.7) from eBioscience, and I-A/I-E (M5/114.15.2) from Biolegend. Propidium iodide (Sigma Aldrich) was used to exclude dead cells. Cells were sorted using a BD FACS Aria (70 µm nozzle, 50 psi, BD Biosciences). After an initial sort to verify purity, a second sort was performed to collect DC populations of 95–100% purity directly into TRIzol reagent (Invitrogen). Sample size for each DC population is as follows: CD4 DCs, n = 5 in spleen, n = 3 in SLN, n = 3 in MLN; CD8 DCs, n = 5 in spleen, n = 3 in SLN, n = 3 in MLN, n = 3 in thymus; pDCs, n = 3 in spleen, n = 3 in SLN, n = 2 in MLN; CD4^−^CD8^−^CD11b^+^ double negative (DN) DCs n = 3 in spleen, n = 3 in SLN, n = 3 in MLN; CD4^−^CD8^−^CD11b^−^ triple negative (TN) DCs, n = 3 in spleen, n = 4 in SLN, n = 5 in MLN.

For flow cytometry analysis, cells were stained in FACS buffer containing FcR-block antibody (2.4G2) with combinations of fluorochrome-conjugated or biotinylated antibodies against CD11b (M1/70), CD4 (L3T4), CD11c (N418), CD8 (53-6.7), CD45 (30.F11), Gr1 (RB6-8C5), CD3ε (145-2C11), NK1.1 (PK136) CD19 (MB19-1), PDCA-1 (eBio927), Tim-1 (RMT1-4) and B220 (RA3-6B2) from eBioscience. Cells were analyzed using a BD FACS Aria and phenotypes were analyzed using FlowJo Software (Tree Star, Inc).

### Detection of aldehyde dehydrogenase activity

Aldehyde dehydrogenase activity was tested using the Aldefluor assay kit (StemCell Technologies) as described in the manufacturer's protocol with some modifications (100 µM of DEAB reagent (Sigma) and 300 nM of the Aldefluor reagent). Aldefluor incubation was followed directly by surface marker staining and flow cytometric analysis.

### RNA isolation and microarray analysis

Total RNA was prepared using chloroform extraction according to manufacturer's protocol, and 100 ng of RNA from each sample was used for amplification, labeling, and hybridization by Expression Analysis, Inc. (Durham, NC). Mouse Gene ST 1.0 chips (Affymetrix) were used for microarray analysis. All data is MIAME compliant and the raw data for the Immunological Genome Project (ImmGen) including dendritic cells analyzed in this study have been deposited in a MIAME compliant database [NCBI Gene Expression Omnibus (GEO) data repository; record no: GSE15907].

Various modules included in the GenePattern platform (Broad Institute) were used for data analysis. Raw data were normalized using the ExpressionFileCreator module (RMA method). The MultiPlot module was used for dataset comparisons including fold change analysis and statistical filtering. In each analysis, we preprocessed the dataset for the populations included in specific analyses. The first criterion was to select probes with mean expression value greater than 120, which gives real signal with 95% confidence. We selected all probes with mean expression above 120 in at least one of the populations analyzed (exceptions: 163 for CD4 MLN, 175 for CD4 SLN, 153 for CD8 SLN, 150 for CD8 SPL, 149 for DN MLN, 172 for DN SPL, and 170 for TN MLN. For simplicity, the value was stated as “120” in the Results and Figure Legends). This filter was used in combination with coefficient of variation (CV)<0.5 for replicates. In all fold change analyses, an arbitrary cutoff value of 2 was used. T-test P values for each probe for >2-fold differences in expression were indicated in supplementary tables and P<0.05 was considered statistically significant. For hierarchical clustering, Spearman's correlation was used with datasets following log2 transformation, row centering (subtraction of the mean of each row), and row normalization (sum of the squares of the values in each row is 1.0) in the HierarchicalClustering module. Heatmaps were constructed using the HeatmapViewer module. Correlation matrices were constructed after preprocessing datasets using the Expression Matrix module created by ImmGen. Dataset for the specific subsets that are analyzed were first filtered for probes with expression >120 for at least one population, CV<0.5 for each population and fold difference >2 for at least two populations. Data was further preprocessed as described for hierarchical clustering. Principal component analyses were done using the PopulationDistances module created by ImmGen. In this analysis, 16 DC subsets were included. First probes with mean expression value >120 were selected, then dataset was log2 transformed row centered (subtraction of the mean of each row), and column centered (division by standard deviation). PCA was computed on the 15% most differentially expressed genes among subsets. In principal component analysis, the first principal component represents 38.06% variation, second principal component represents 23.95% variation, and the third principal component represents 16.17% variation. The purity of sorted samples was validated by comparing expression values of various genes (*Cd4*, *Cd8a*, *Itgam*, *Itgax*, *Itga2*, *Ncr1*, *Klrb1c*, *Cd3e*, *Cd247*, *Cd8b*, *Cd19*, *Cd79a*, *Siglech*, *Ly6g* and *Ly6c*) to other immune cell subsets included in the ImmGen dataset (Ref. [50] data not shown).

### Statistical analysis for non-microarray data

Data in bar graphs are presented as the mean±SD and were analyzed using the one-tailed, unpaired Student's T-test for comparison of two groups. *P* values <0.05 were considered statistically significant.

## Supporting Information

Figure S1 **Strategy for sorting DC subsets from primary and secondary lymphoid organs.** For high purity sorting, conventional DCs were sorted from CD3ε/CD19/NK1.1/Gr1/Ter119-depleted cells, while pDCs were sorted from CD3ε/CD19/NK1.1/Ter119-depleted cells. (A) Representative plots showing the gating strategy used for sorting and post-sort purity analysis of CD4 (CD11c^hi^CD11b^+^CD4^+^CD8^−^, n = 5 in SPL, n = 3 in SLN, n = 3 in MLN), CD8 (CD11c^hi^CD11b^−^CD4^−^CD8^+^, n = 5 in SPL, n = 3 in SLN, n = 3 in MLN), CD4^−^CD8^−^CD11b^+^ (CD11c^hi^CD11b^+^CD4^−^CD8^−^, n = 3 in SPL, n = 3 in SLN, n = 3 in MLN) double negative (DN) DCs, and CD4^−^CD8^−^CD11b^−^ (CD11c^hi^CD11b^−^CD4^−^CD8^−^, n = 3 in SPL, n = 4 in SLN, n = 5 in MLN) triple negative (TN) DCs from the SPL (top), SLN (middle) and MLN (bottom). (B) Representative plots showing the gating strategy used for sorting and post-sort purity analysis of thymic CD8 DCs (MHC-II^hi^CD11c^hi^CD8^+^CD11b^−^, n = 3). (C) Representative plots showing the gating strategy used for sorting and post-sort purity analysis of pDCs (CD11c^int^B220^+^Gr1^+^CD8^+^, n = 3 in SPL, n = 3 in SLN, n = 2 in MLN) from the SPL (top), SLN (middle) and MLN (bottom).
(TIF)

Figure S2 **Expression values of various genes associated with DCs and myeloid cells.** Bar graphs showing the expression value of *Met*, *Ppap2a*, *Dscam*, *Duxbl* and *Slamf9* for CD8 DCs, CD4 DCs, DN DCs, TN DCs and pDCs from different lymphoid organs and naïve NK cells, CD4 T cells, CD8 T cells and B cells from spleen (data obtained from ImmGen).
(TIF)

Table S1 **Canonical gene expression profile of CD8 DCs, CD4 DCs and pDCs across lymphoid organs.**
(XLS)

Table S2 **Probes associated with CD8 DCs, CD4 DCs or pDCs across lymphoid organs; gene list based on 2-fold change in one subset compared to the other two in spleen.**
(XLS)

Table S3 **Probes associated with CD8 DCs, CD4 DCs or pDCs in a given lymphoid organ.**
(XLS)

Table S4 **Combination of genes identified in CD8 and CD4 DCs across lymphoid organs ordered according to hierarchical clustering in heatmap.**
(XLS)

Table S5 **Comparison of CD8 DCs across secondary lymphoid organs (SLO) based on thymus and spleen associated probes.**
(XLS)
