# Supplementary figures and images for: Lymphoid Organ-Resident Dendritic Cells Exhibit Unique Transcriptional Fingerprints Based on Subset and Site
Source: PLoS One. 2011 Aug 19;6(8):e23921. doi: 10.1371/journal.pone.0023921 (PMC3158776; doi:10.1371/journal.pone.0023921)

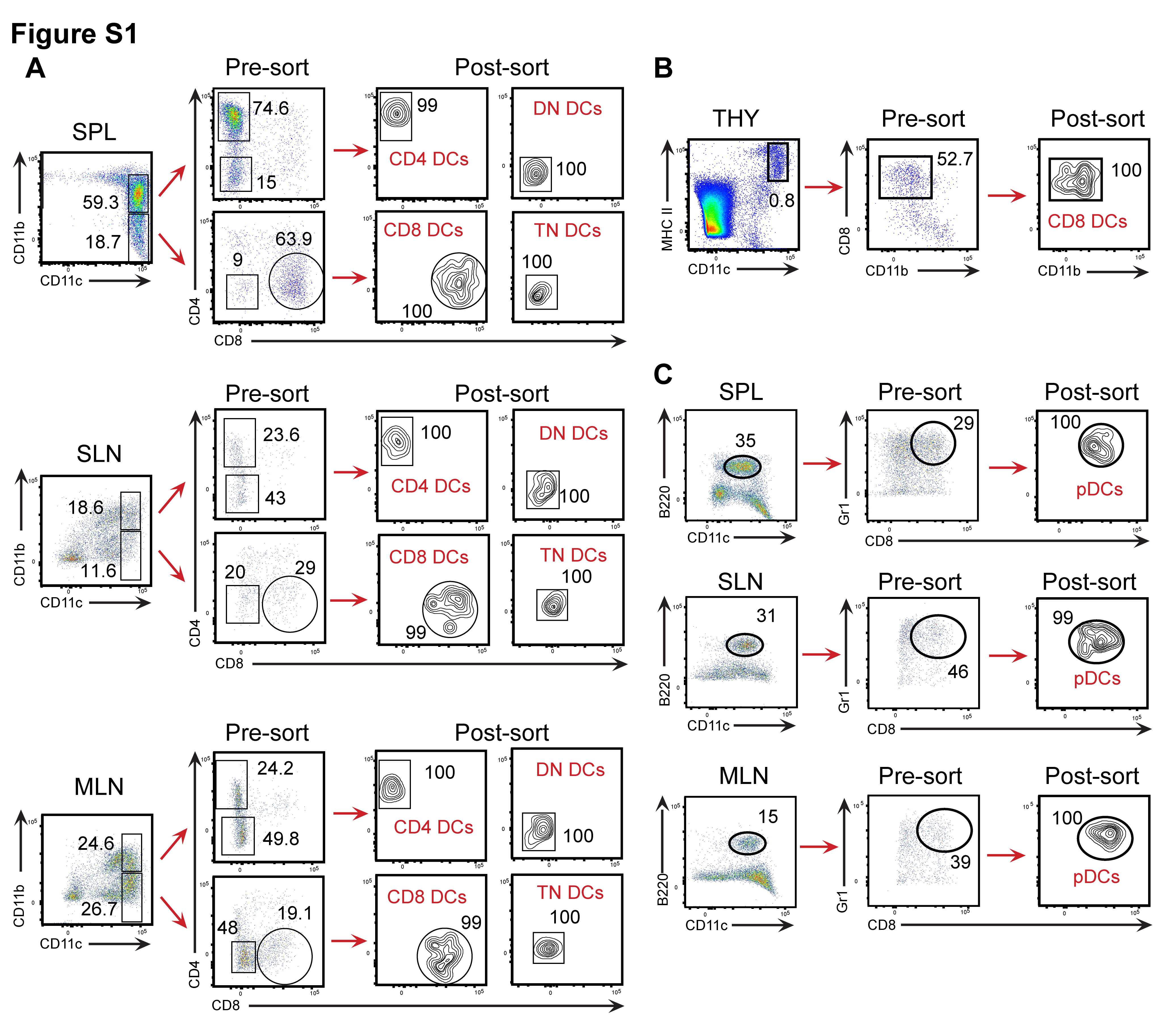

Supplement: Figure S1 — Strategy for sorting DC subsets from primary and secondary lymphoid organs. For high purity sorting, conventional DCs were sorted from CD3ε/CD19/NK1.1/Gr1/Ter119-depleted cells, while pDCs were sorted from CD3ε/CD19/NK1.1/Ter119-depleted cells. (A) Representative plots showing the gating strategy used for sorting and post-sort purity analysis of CD4 (CD11chiCD11b+CD4+CD8−, n = 5 in SPL, n = 3 in SLN, n = 3 in MLN), CD8 (CD11chiCD11b−CD4−CD8+, n = 5 in SPL, n = 3 in SLN, n = 3 in MLN), CD4−CD8−CD11b+ (CD11chiCD11b+CD4−CD8−, n = 3 in SPL, n = 3 in SLN, n = 3 in MLN) double negative (DN) DCs, and CD4−CD8−CD11b− (CD11chiCD11b−CD4−CD8−, n = 3 in SPL, n = 4 in SLN, n = 5 in MLN) triple negative (TN) DCs from the SPL (top), SLN (middle) and MLN (bottom). (B) Representative plots showing the gating strategy used for sorting and post-sort purity analysis of thymic CD8 DCs (MHC-IIhiCD11chiCD8+CD11b−, n = 3). (C) Representative plots showing the gating strategy used for sorting and post-sort purity analysis of pDCs (CD11cintB220+Gr1+CD8+, n = 3 in SPL, n = 3 in SLN, n = 2 in MLN) from the SPL (top), SLN (middle) and MLN (bottom). (TIF) [file pone.0023921.s001.tif]

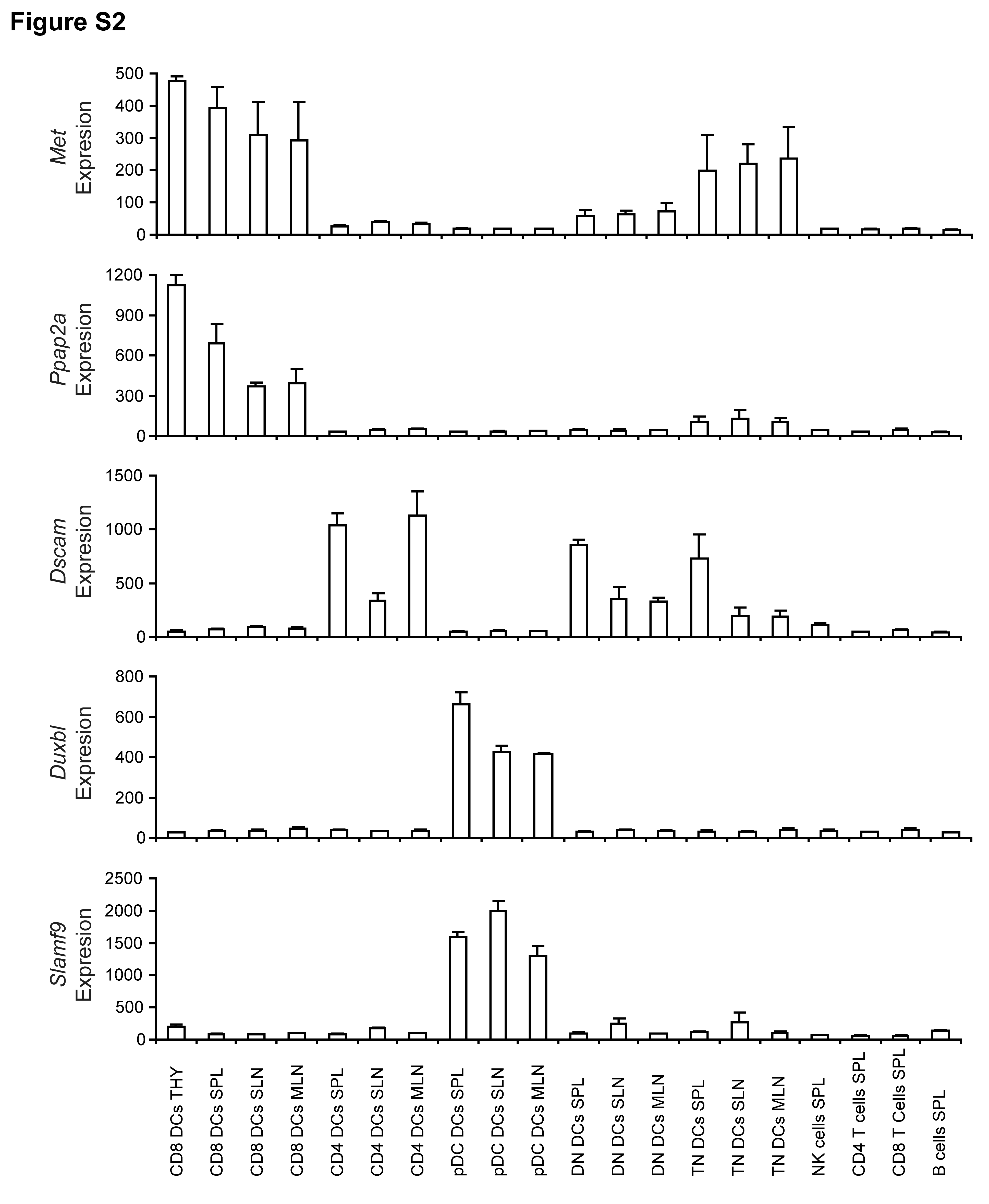

Supplement: Figure S2 — Expression values of various genes associated with DCs and myeloid cells. Bar graphs showing the expression value of Met, Ppap2a, Dscam, Duxbl and Slamf9 for CD8 DCs, CD4 DCs, DN DCs, TN DCs and pDCs from different lymphoid organs and naïve NK cells, CD4 T cells, CD8 T cells and B cells from spleen (data obtained from ImmGen). (TIF) [file pone.0023921.s002.tif]
